# The protective role of selenium against high-fructose corn syrup–induced kidney damage: a histopathological and molecular analysis

**DOI:** 10.1007/s00210-024-03149-w

**Published:** 2024-05-11

**Authors:** Muhammet Yusuf Tepebaşı, Mehtap Savran, Samet Coşan, Şerife Ağırca Taştan, Bünyamin Aydın

**Affiliations:** 1 Department of Medical Genetics, University of Süleyman Demirel, Isparta, 32300 Turkey; 2 Department of Medical Pharmacology, University of Süleyman Demirel, Isparta, Turkey; 3 Department of Patology, University of Mehmet Akif Ersoy, Burdur, Turkey; 4 Department of Internal Medicine, Kütahya University of Health Sciences, Kütahya, Turkey

**Keywords:** Apoptosis, Endoplasmic reticulum stress, High-fructose diet, Kidney, Rats, Selenium

## Abstract

With the growth of the food industry, fructose, the intake of which increases with food, causes obesity and metabolic syndrome. Kidney damage may develop from metabolic syndrome. Selenium (Se) participates in the structure of antioxidant enzymes and has a medicinal effect. In this work, the protective impact of Se on kidney damage produced by high-fructose corn syrup (HFCS) via endoplasmic reticulum (ER) stress was examined. The study comprised four groups, each consisting of ten experimental animals: control, HFCS (20%-HFCS), HFCS (20%-HFCS), + Se (0.3 mg/kg/day/po), and Se (0.3 mg/kg/day/po) alone. The duration of the experiment was 6 weeks. Kidney tissues were stained with hematoxylin and eosin for histological examination. Immunohistochemical analysis was conducted to assess TNF-α and caspase-3 levels. The spectrophotometric evaluation was performed to measure TOS (total oxidant status), TAS (total antioxidant status), and OSI (oxidative stress index) levels. The PERK, ATF4, CHOP, BCL-2, and caspase-9 gene expression levels were assessed by the RT-qPCR method. After Se treatment, histopathological abnormalities and TNF-α and caspase-3 levels in the HFCS+Se group decreased (*p* < 0.001). While TOS and OSI levels increased dramatically in the HFCS group, TAS values decreased significantly but improved after Se application (*p* < 0.001). The expression levels of the genes PERK, ATF4, CHOP, and caspase-9 were significantly lower in the HFCS group when compared to the HFCS+Se group (*p* < 0.05). Our findings suggest that Se may protect against ER stress, oxidative stress, apoptosis, and kidney damage caused by high-dose fructose consumption.

## Introduction

High-fructose corn syrup (HFCS) is made by isomerizing glucose from the hydrolysis of corn starch into fructose, a highly lipogenic sugar. It is widely used in the food industry because it increases the shelf life of foods, is inexpensive, and plays an important role in the modern diet (Abdel-Kawi et al. 2016; Seylam et al. 2021). Although fructose and glucose are similar molecules, fructose’s ability to bypass the checkpoint of inhibitory signals from enzymatically controlled citrate and ATP allows glycolysis to proceed independent of energy needs, allowing all central functions to proceed, including lipogenesis and oxidative phosphorylation, and providing an increased substrate for carbon metabolic pathways (Korkmaz 2008; Smeraglio et al. 2013; Örek et al. 2020).

Previous research has shown that eating more fructose and exercising less can lead to obesity, liver disease, hypertension, metabolic syndrome, and type 2 diabetes (Ahangarpour et al. 2012; Yeşilot et al. 2022). According to reports, diseases may develop as a result of metabolic syndrome and cause kidney damage (Kurella et al. 2005; Abdel-Kawi et al. 2016). High fructose consumption has been shown in animal studies to cause kidney damage (Kizhner and Werman 2002; Aoyama et al. 2012; Oudot et al. 2013). Increased fructose uptake promotes lipogenesis and lipid accumulation in adipose tissue, resulting in increased release of inflammatory mediators such as tumor necrosis factor-α (TNF-α) and reactive oxygen species (ROS) (Cave et al. 2007). Furthermore, it has been established that an increase in fructose catabolism contributes to lipid peroxidation by increasing ROS (Punitha et al. 2005; Reddy et al. 2009; Bratoeva et al. 2017).

The endoplasmic reticulum (ER), peroxisomes, lysosomes, and mitochondria all experience oxidative stress brought on by naturally occurring ROS; however, this is often controlled by the antioxidant enzyme system in cells (Small et al. 2012). The formation of disulfide bonds generates a large amount of ROS in the ER. Moreover, increased electron transport system activity brought on by energy requirements for chaperone and ER protein functions raises mitochondrial ROS generation and oxidative stress in pathological processes like ER stress (Lenaz 2001; Herst et al. 2017). Increased ER stress can activate apoptotic caspase-3 via changes in C/EBP homologous protein (CHOP) and B cell lymphoma 2 (BCL-2) expression (Suganya et al. 2014). Moreover, elevated ER stress may activate nuclear factor erythroid 2–related factor 2 in response to oxidative stress by upregulating the expression of genes involved in the PERK/eIF2/ATF4 pathway (Jin et al. 2017).

Selenium (Se), a structural component of antioxidant enzymes such as glutathione peroxidases and thioredoxin reductase, increases antioxidant activity and plays a role in protecting cells and tissues against damage caused by oxidative stress (Letavayová et al. 2008; Liu et al. 2015a). The curative effect of Se on ER stress and apoptosis was determined as a result of changes in the expression of the genes involved in the activating transcription factor 4 (ATF)–ATF6 increase and apoptosis (caspase-3 increase-BCL-2 decrease) in chicken liver in Se deficiency (Yao et al. 2015). In addition, in a study on rat kidneys, it was found that Se showed a protective effect on toxicity by reducing oxidative stress, ER stress, and apoptosis through caspase-3, GPx, Bax, SOD, caspase-12, Bip, CHOP, c-Jun N-terminal kinase (JNK), and BCL-2 genes (Zhang et al. 2020). It has been shown that Se shows its antioxidant effect by decreasing malondialdehyde (MDA) and increasing superoxide dismutase (SOD), reducing ER stress by decreasing glucose-regulated protein 78 (GRP78), GRP94, ATF4, ATF6, and inositol-requiring enzyme (IRE) gene expressions, and its anti-apoptotic effect by causing a decrease in caspase-3 gene expression levels. It has been determined that Se has a protective effect on nephrotoxicity in these ways (Liu et al. 2015b). Selenium can regulate the activity of the eicosanoid synthesis pathway, leading to leukotriene and prostacyclin synthesis and downregulation of the expression of cytokines and adhesion molecules. Selenoprotein S also functions to mediate immune responses and reduce endoplasmic reticulum stress in peripheral macrophages. Due to these properties, Se may have anti-inflammatory effects on the kidneys (Liu et al. 2021).

The purpose of this study was to determine whether Se had any protective effects on the cellular and histopathologic mechanisms of inflammation, oxidative stress, ER stress, and apoptotic cell death brought on by the HFCS diet in rat kidneys. There are not enough studies on the curative effect of Se in healing the damage caused by fructose on kidney tissue. For this purpose, we investigated the curative effect of Se given at doses of 0.3 mg/kg on kidney damage induced by HFCS for 6 weeks, with histological (caspase-3 and TNF-α), biochemical (oxidative stress markers), and PCR analyses (protein kinase R (PKR)–like endoplasmic reticulum kinase (PERK), ATF, CHOP, BCL-2, and caspase-9).

## Materials and methods

### Animals and experiment protocol

The experiment included four groups, each comprising ten male Wistar albino rats weighing between 250 and 350 g, and lasted for 6 weeks.

Control group (*n* = 10): Rats received regular drinking water without any additives for the entire 6-week period.

HFCS group (*n* = 10): Rats were provided with drinking water containing a 20% mixture of a 55% fructose solution for 6 weeks (İlhan et al. 2024).

HFCS and Se group (*n* = 10): Following 6 weeks of introducing the 55% solution at a 20% rate into the feeding water, 0.3 mg/kg/day/po Se was given through drinking water (Abd Al Haleem and El-Bakly 2019).

Se group (*n* = 10): 0.3 mg/kg/day/po for 6 weeks, Se was infused into drinking water.

The experimental animals were put to death under anesthesia after 6 weeks. Following sacrification, the kidney’s tissues were stored in formaldehyde for histological examination. The leftover kidney tissues were stored for genetic and biochemical studies at −80 °C.

### Histopathological analysis

Whether there was any pathological finding in the kidney tissues during necropsy was examined macroscopically, and it was fixed in 10% formalin solution. After fixation, Leica ASP300S automatic tissue processing equipment was used to process the tissue samples and embed them in paraffin wax. Following cooling, microtomes (Leica Microsystems, Wetzlar, Germany) were cut at 5 μm from the paraffin blocks. Following hematoxylin-eosin (H&E) staining, the sections were viewed under a light microscope.

The histopathological lesions in the tissues were assessed semiquantitatively using an ordinal grading scheme. Hyperemia, hemorrhage, inflammatory cell infiltrations, and degenerative and necrotic alterations were all investigated. The descriptions of normal (score = 0), mild (score = 1), moderate (score = 2), and severe (score = 3) affections were given scores ranging from 0 to 3 (Ilhan et al. 2024).

### Immunohistochemical analysis

Furthermore, two series of sections from all blocks drawn on poly-L-lysine coated slides were immunohistochemically stained for caspase-3 (anti-caspase-3 antibody [EPR18297], ab184787)) and TNF-α (anti-TNF alpha antibody [TNFA/1172], ab220210)) expression using the streptavidin-biotin technique according to manufacturer instructions. All primary antibodies from Abcam (Cambridge, UK) were used at a dilution of 1/100. After 60 min of incubation with the primary antibodies, the sections were immunohistochemically stained with a secondary antibody. The Expose Mouse and Rabbit Specific HRP/DAB Detection IHC Kit (ab80436, Abcam, Cambridge, UK) included the secondary antibody. As a chromogen, diaminobenzidine (DAB) was employed. For negative controls, antigen dilution solution was utilized in place of the main antibody. In addition, the samples were analyzed by another pathologist as blind samples.

During the immunohistochemistry examination, each antibody-specific section was examined independently. A grading system from 0 to 3 was used in a semiquantitative analysis to assess the level of immunohistochemistry reactivity in cells containing markers. For each slice, ten distinct areas were examined under a 40× objective magnification for assessment. The Database Manual Cell Sens Life Science Imaging Software System (Olympus Co., Tokyo, Japan) was utilized for morphometric analysis and microphotography.

### Biochemical analyses

Kidney tissues from rats, weighing approximately 150 mg each, were homogenized using the Ultra Turrax Janke & Kunkel T-25 homogenizer (IKA® Werke, Germany) with a 1:9 (w/v) ratio of phosphate-buffered saline (PBS). After homogenization, the samples were centrifuged at 10,000 rpm for 10 min to extract oxidative stress markers from the kidney tissue. Using an automated analyzer equipped (Beckman Coulter, USA) with Erel’s colorimetric approach, the levels of oxidative stress index (OSI), total oxidant status (TOS), and total antioxidant status (TAS) in samples were assessed (Erel 2005). The OSI value was calculated as [(TOS, µmol/l) / (TAS, mmol Trolox eq/l) × 100] (Altindag et al. 2008).

### Real-time qPCR analysis

Following the manufacturer’s instructions, RNA was isolated from homogenized kidney tissues using the Geneall Ribospin RNA isolation kit. RNA samples were quantified in terms of both amount and purity using a nanodrop technology (Thermo Fisher Scientific, USA). For cDNA synthesis, the isolated RNA samples were stored at −80 °C and standardized to 500 ng/µl. The A.B.T. TM cDNA Synthesis Kit (Atlas Biotechnology, Turkey) methodology was used for cDNA synthesis, which was completed in a thermal cycler (Thermo Fisher Scientific, USA) with a total reaction volume of 20 for each sample. Primer designs were generated by identifying specific mRNA sequences and evaluating potential primer sequences on the NCBI website. Table 1 lists the specific primer sequences and genes used in the expression stage. The Biorad CFX96 (CA, USA) real-time qPCR instrument was used to measure the expression levels using the SYBR-Green MasterMix (Atlas Biotechnology, Turkey). As per the manufacturer’s protocol, 40 cycles of 20 s at 95 °C and 30 s at 60 °C were conducted for RT-qPCR. Pre-denaturation was carried out at 95 °C for 5 min. Actin B gene expression was used as the standard, and each sample was analyzed in triplicate.

**Table 1:** Primers and product sizes for real-time qPCR assay

Gene	Specific primer sequence	Product length
Actin B (HouseKeeping)	F: CATGTACGTTGCTATCCAGGC	250 bp
	R: CTCCTTAATGTCACGCACGAT	
PERK	F: CCAAGCTGTACATGAGCCCAGA	178 bp
	R: TTCTGAGTGAACAGTGGTGGAAAC	
ATF4	F: AGTCTGCCTTCTCCAGGTGTTC	134 bp
	R: GCTGTCTTGTTTTGCTCCATCTT	
CHOP	F: TGGAAGCCTGGTATGAGGATCTG	175 bp
	R: GAGGTGCTTGTGACCTCTGCTG	
BCL-2	F: ATCGCTCTGTGGATGACTGAGTAC	134 bp
	R: AGAGACAGCCAGGAGAAATCAAA	
Caspase-9	F: AGCCAGATGCTGTCCCATAC	148 bp
	R: CAGGAACCGCTCTTCTTGTC	

*PERK*, PRKR-like endoplasmic reticulum kinase; *ATF4*, activating transcription factor 4; *CHOP*, C/EBP homologous protein; *BCL-2*, B cell lymphoma 2; *caspase-9*, cysteine-aspartic acid protease

### Statistical analysis

Statistical analyses of the scoring of histopathological changes were performed with the Pearson chi-square test. Normality distributions were evaluated with the Kolmogorov-Smirnov test. The Kruskal-wallis one-way ANOVA Dunn-Bonferroni test was used to determine the difference between groups and median-quartile values were stated. SPSS-20.00 (SPSS Inc., Chicago, IL) software was used for all statistical analyses. *p* < 0.05 was considered significant.

## Results

### Histopathological results

**Fig. 1:**
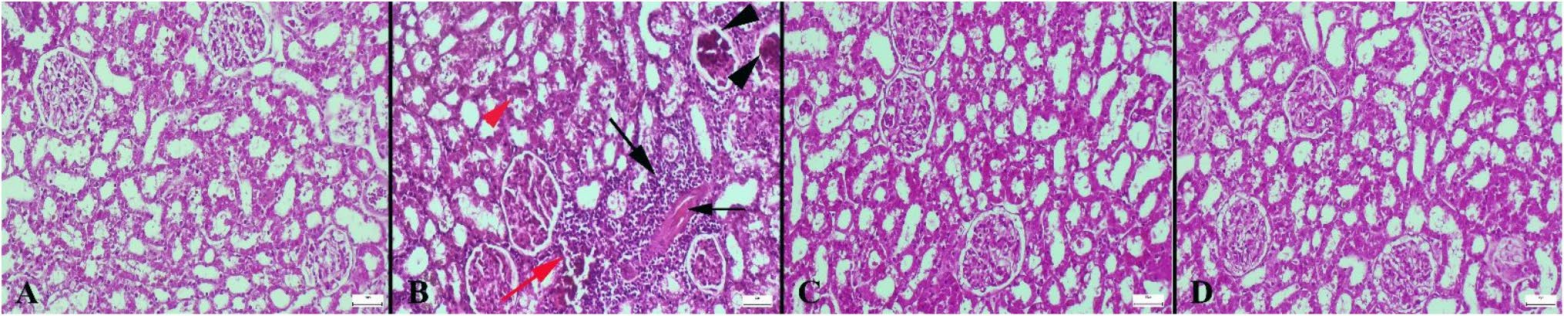
Kidney histopathological images in each group. **A** The kidney histology of the control group was normal. **B** Marked hyperemia (thin black arrow), micro hemorrhage (red arrow), inflammatory cell infiltrations (black thick arrow), proteinous materials and sclerosis in the glomerulus (black arrowheads), and necrotic cell clusters in the lumen of a tubule (red arrowhead) were observed in the HFCS group. **C** Pathological results were reduced in the HFCS+Se group. **D** The Se group showed a typical tissue structure, H&E, scale bar = 50 μm

In the control and Se groups, normal kidney histoarchitecture was observed during the histological examination. The HFCS group displayed marked hyperemia, micro hemorrhages, and mild to severe inflammatory cell infiltration. Additionally, cystic dilatations in tubules, proteinous material in the tubular lumen, and glomerulus were common findings. Microthrombosis in some capillaries was rarely observed. Vacuolar degeneration and desquamation are generally noticed in the tubular epithelial cells of the HFCS group. In the HFCS+Se group, Se therapy significantly reduced pathological findings (Fig. 1) (Table 2).

**Table 2:** Histopathological evaluation and statistical analysis between groups

Evaluation criteria	Grading scores	Control (*N*) (%)	HFCS (*N*) (%)	HFCS+Se (*N*) (%)	Se (*N*) (%)	*X*^2^	*p*-value
Hyperemia	0	7 (87.5)^a^	0 (0)^b^	5 (62.5)^a^	8 (100)^a^	20.600	0.002^*^
	1	1 (12.5)^a^	5 (62.5)^b^	2 (25)^a^	0 (0)^a^		
	2	0 (0)^a^	3 (37.5)^b^	1 (12.5)^a^	0 (0)^a^		
Micro hemorrhage	0	8 (100)^a^	0 (0)^b^	6 (75)^a^	8 (100)^a^	26.390	< 0.001^*^
	1	0 (0)^a^	5 (62.5)^b^	2 (25)^a^	0 (0)^a^		
	2	0 (0)^a^	3 (37.5)^b^	0 (0)^a^	0 (0)^a^		
Inflammatory cell infiltrations	0	8 (100)^a^	0 (0)^b^	5 (62.5)^a^	8 (100)^a^	24.343	< 0.001^*^
	1	0 (0)^a^	7 (87.5)^b^	3 (37.5)^a^	0 (0)^a^		
	2	0 (0)^a^	1 (12.5)^b^	0 (0)^a^	0 (0)^a^		
Degeneration	0	8 (100)^a^	4 (50)^b^	7 (87.5)^a^	8 (100)^a^	10.193	0.017^*^
	1	0 (0)^a^	4 (50)^b^	1 (12.5)^a^	0 (0)^a^		
	2	0 (0)^a^	0 (0)^b^	0 (0)^a^	0 (0)^a^		

*N*, number; *%*, percentage; *HFCS*, high-fructose corn syrup; *Se*, selenium. The differences between the groups carrying different letters in the same row are statistically significant, ^*^p-value < 0.05 is considered statistically significant

### Immunohistochemical findings

**Fig. 2:**
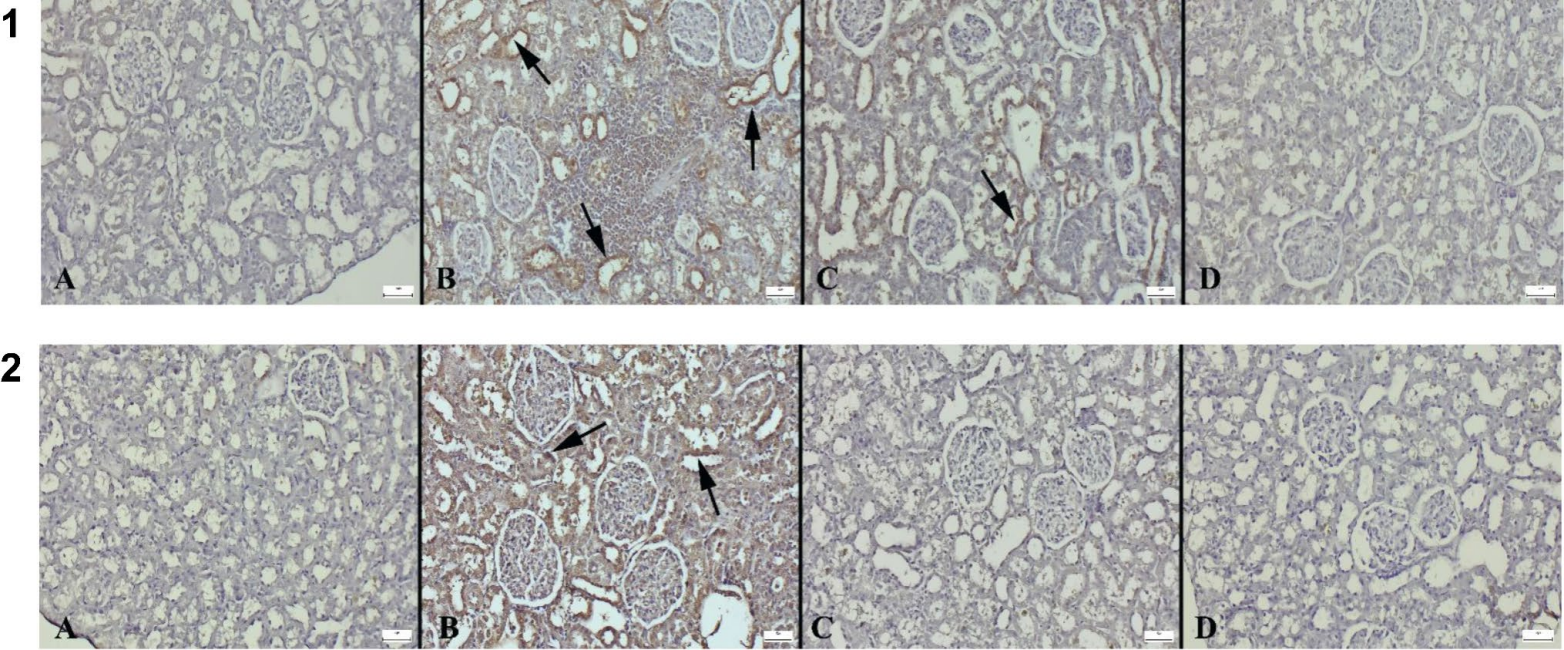
(1) Results of caspase-3 immunohistochemistry comparing the groups. (A) Negative expression observed in the control group. (B) A marked increase in expression (arrows) in the HFCS group. (C) Decreased expression in the HFCS+Se group. (D) No expression was observed in the Se group, streptavidin-biotin peroxidase method, scale bar = 50 μm. (2) Results of TNF-α immunohistochemistry comparing the groups. (A) No expression was observed in the control group. (B) The tubular cells of the HFCS group showed a noticeably higher level of expression (arrows). (C) The HFCS+Se group showed decreased expression. (D) No expression was observed in the Se group, streptavidin-biotin peroxidase technique, scale bar = 50 μm

Immunohistochemical analysis showed that the HFCS group had higher caspase-3 expression. Se treatment reduced the caspase-3 expression in the HFCS+Se. Expressions were most frequently observed in kidney tubular cells. In the control and Se groups, expressions ranging from slight to negative were observed (Table 3) (Fig. 2).

**Table 3:** Statistical analysis results of immunohistochemical parameters between the groups

	Control	HFCS	HFCS+Se	Se	*p*-value
Caspase-3	0 (0.00–0.75)	2.00 (2.00–2.75)^b^	0.50 (0.00–1.00)^a^	0.00 (0.0–0.0)^a^	< 0.001
TNF-α	0.00 (0.0–0.0)^a^	1.00 (1.00–2.00)^b^	0.00 (0.0–1.00)^a^	0.00 (0.0–0.0)^a^	< 0.001

*HFCS*, high-fructose corn syrup; *Se*, selenium; *TNF-α*, tumor necrosis factor-alpha. Data were expressed as median (IQR) and 25–75% percentile. The differences between the groups carrying different letters in the same row are statistically significant, *p* < 0.001

During microscopic examination of TNF-α immunostained sections, the control and Se groups showed negative expression, whereas the HFCS group showed increased expression. Expressions in the HFCS+Se group decreased as a result of Se therapy (Table 3) (Fig. 2).

This investigation demonstrates that HFCS caused kidney damage and that Se has a protective effect against HFCS-induced damage.

### Biochemical analysis results

The effects of corn syrup on oxidative stress were assessed using kidney-homogenized tissue samples and TOS, TAS, and OSI parameters. The corn syrup group’s TOS and OSI values were much higher than those of the control group. However, TAS values were significantly lower (*p* < 0.001, *p* < 0.001, and *p* < 0.001, respectively). The TOS and OSI values decreased while the TAS values sharply increased when we compared the HFCS group to the HFCS+Se and Se groups (Table 4). According to the findings, HFCS-induced oxidative stress decreased with the addition of Se.

**Table 4:** Statistical comparison of TOS, TAS, and OSI values between groups

	Control	HFCS	HFCS+Se	Se	*p*-value
TOS (µmol/l)	12.26 (7.85–12.84)	15.44 (14.48–17.54)^a^	11.20 (9.73–12.07)^b^	11.21 (10.31–12.58)^c^	*p* < 0.001^a^ *p* = 0.002^b^ *p* = 0.002^c^
TAS (mmol Trolox eq/l)	1.56 (1.51–1.65)	1.36 (1.25–1.40)^b^	1.54 (1.50–1.60)^b^	1.59 (1.50–1.67)^c^	*p* < 0.001^a^ *p* = 0.017^b^ *p* < 0.001^c^
OSI (%)	0.76 (0.50–0.84)	1.15 (1.08–1.21)^b^	0.74 (0.66–0.76)^b^	0.70 (0.62–0.84)^c^	*p* < 0.001^a^ *p* < 0.001^b^ *p* < 0.001^c^

Data were expressed as median (IQR) and 25–75% percentile. ^a^Control vs. HFCS, ^b^HFCS vs. HFCS+Se, ^c^HFCS vs. Se. *HFCS*, high-fructose corn syrup; *Se*, selenium; *TOS*, total oxidant status; *TAS*, total antioxidant status; *OSI*, oxidative stress index; *SD*, standard deviation

### RT-PCR analysis results

**Fig. 3:**
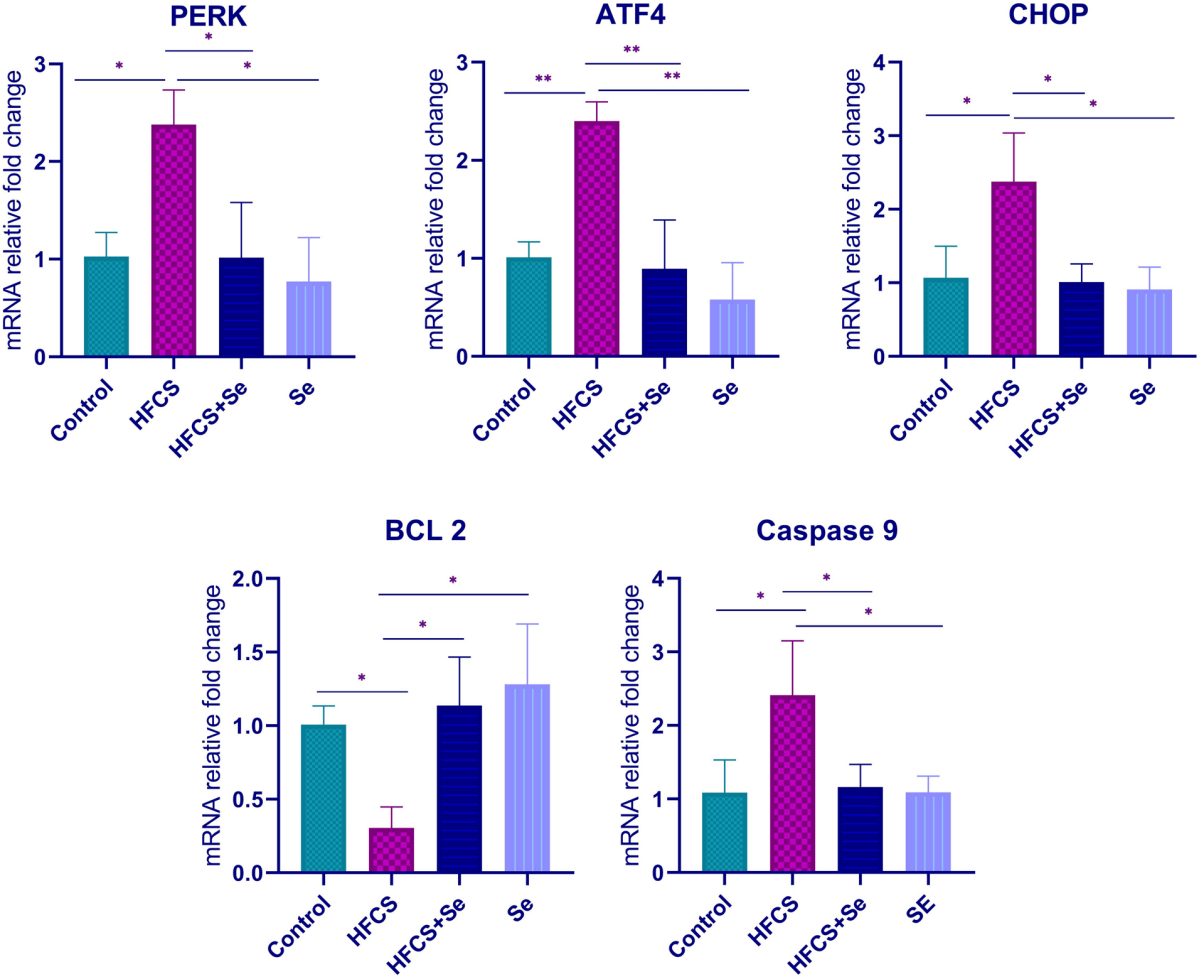
Values of ER stress and mitochondrial apoptosis genetic parameters were given as mean ± SD, and a one-way ANOVA test evaluated the results of relative mRNA expression. HFCS, high-fructose corn syrup; Se, selenium; PERK, protein kinase R (PKR)–like endoplasmic reticulum kinase; ATF4, activating transcription factor 4; CHOP, C/EBP homologous protein; BCL-2, B cell lymphoma 2; caspase-9, cysteine-aspartic acid protease 9. *p  < 0.05, **p  < 0.001

The relative fold changes of mRNA of ER stress pathway genes PERK, ATF4, CHOP, anti-apoptotic BCL-2, and proapoptotic caspase-9 genes were compared between groups. Comparing the HFCS group to the control group revealed that the HFCS group had lower BCL-2 gene expression and higher expressions of PERK, ATF4, CHOP, and caspase-9. The expression levels of the PERK, ATF4, CHOP, and caspase-9 genes were significantly lower in the HFCS group when compared to the HFCS+Se group, but the expression level of the BCL-2 gene was statistically significantly higher. When the control and HFCS groups were compared, it was determined that PERK, ATF4, CHOP, and caspase-9 expression increased 2.3, 2.4, 2.2, and 2.2 fold, respectively, and BCL-2 expression decreased 3.4 times in the HFCS group. When the HFCS and HFCS+Se groups were compared, it was determined that PERK, ATF4, CHOP, and caspase-9 expression decreased 2.3, 2.7, 2.3, and 1.5 fold, respectively, and BCL-2 expression increased 3.8 times in the HFCS+Se group. Figure 3 also depicts mRNA relative fold change graphs for genes findings showed that Se alleviated the HFCS-induced ER stress and apoptosis.

## Discussion

Our research demonstrated that the increase in the proinflammatory marker TNF-α and the histologically observed modest infiltration of inflammatory cells in the kidney tissue exacerbated inflammation in rats receiving HFCS. Additionally, we investigated the effects of increased fructose consumption on rat kidneys. We found a significant decrease in TAS levels, while TOS and OSI increased in the kidneys of HFCS-treated rats. When we evaluated ER stress through the PERK, ATF4, and CHOP pathways, there was a significant increase in the expression levels of these genes. We demonstrated that HFCS decreases BCL-2 and increases caspase-3 and caspase-9 in the apoptotic process. In addition, we found histopathological changes supported these data. This evidence revealed that high-fructose-related nutrition can cause damage in rat kidneys by increasing oxidative stress, ER stress, and apoptosis. Hu et al. reported that a high-fructose diet increased inflammation and oxidative stress by increasing interleukin 6, TNF-α, and NADPH oxidase 2 in rat kidneys. Yeşilot et al. (2022) reported that TNF-α and MDA increased, while catalase decreased in rat kidneys given HFCS. It was also determined that TNF-α increased in the masseter muscles of other HFCS-given rats (Ekici et al. 2022).

Oxidative stress arises when ROS accumulate within the cell. This accumulation can be triggered by various factors, including proinflammatory mediators. In turn, these mediators can further stimulate ROS production, leading to a vicious cycle of oxidative stress. Additionally, ER stress can exacerbate oxidative stress by contributing to an increase in ROS levels (Reyes-Fermín et al. 2020). Moreover, oxidative stress can contribute to kidney diseases by adversely affecting the function of glomerular and tubulointerstitial cells, particularly through impairment of ER function following lipid peroxidation (Inagi et al. 2014). Following ER stress, there is an upregulation in the expression of PERK, ATF4, and CHOP genes within this pathway. Elevated CHOP levels subsequently lead to the downregulation of the BCL-2 gene, resulting in increased expression of caspase-9 and caspase-3 genes. These genes play pivotal roles in the apoptotic process, ultimately leading to cell apoptosis (Suganya et al. 2014). Wang et al. (2019) reported that excessive ER stress in the kidneys induces apoptosis through the activation of the PERK-eIF2α-ATF4-CHOP pathway. Unsal et al. (2020) reported that HFCS elevated the ER and oxidative stress. Furthermore, it has been demonstrated that HFCS promotes apoptosis by raising inflammation and oxidative stress in many organs and tissues (Savran et al. 2019; Topsakal et al. 2019). In conclusion, our literature findings supported the damage caused by HFCS in the kidney.

Through its role in the synthesis of antioxidant enzymes such as thioredoxin reductase and glutathione peroxidases, Se is a trace element that can help reduce oxidative stress (Letavayová et al. 2008). We examined the potential therapeutic benefit of Se on the kidneys of the HFCS+Se given group in this investigation, taking into account its known antioxidant properties. Following the application of Se, we noticed a significant drop in TNF-α levels and an improvement in histological inflammatory cell infiltrations. These results indicate a reduction in the inflammatory effects of HFCS on rat kidneys by Se supplementation. Moreover, in the HFCS+Se group, TAS levels increased, while TOS and OSI decreased. Furthermore, we observed a decrease in the expression of PERK, ATF4, CHOP, caspase-3, and caspase-9, which are involved in the ER stress and apoptosis pathways, along with a significant increase in the anti-apoptotic BCL-2. These findings suggest that Se supplementation alleviates oxidative stress, ER stress, and apoptosis. Additionally, these results were corroborated by histopathological evaluation.

Due to the presence of antioxidant enzymes in the structure and antioxidant properties of Se, studies have determined that it reduces the level of TNF-α in kidney tissue. Al-Brakati et al. (2021) on rat kidneys of Se reported that it has a renoprotective effect with anti-inflammatory and anti-apoptotic effects by increasing BCL-2 levels by decreasing interleukin (IL)-1β, caspase-3, TNF-α, IL-6, and Bax levels. According to Lai et al. (2021), Se deprivation raises apoptosis and inflammation in connection to the SIRT1/PGC1α pathway. Se lowered autophagy and oxidative stress in chicken testicles, according to Huang et al. (2019). Selenium deficit promotes oxidative stress and causes ER stress and apoptosis via the RP78, GRP94, ATF4, ATF6, IRE, caspase-3, and BCL-2 pathways, according to research by Yao et al. (2015). Wan et al. (2018) reported that Se reduces ER stress, apoptosis, and oxidative stress, in chicken ovaries. Reductions in MDA, ATF4, caspase-3, GRP78, ATF6, and IRE are how this impact is attained. In addition, in our previous study on the therapeutic effect of Se, we determined that it has antioxidant, anti-apoptotic, and anti-inflammatory effects on cardiotoxicity (Ilhan et al. 2023).

Our research indicates that Se has the potential to mitigate kidney damage resulting from high-dose fructose intake by modulating TNF-α levels, oxidative stress parameters (TOS, TAS, OSI), ER stress via the PERK/CHOP pathway, apoptosis markers (caspase-3, caspase-9, and BCL-2), and histopathological examination.

## Conclusion

HFCS is commonly utilized to prolong the shelf life of products, yet its escalating consumption has been associated with various disorders, particularly obesity and metabolic syndrome. Our investigation focused on the potential of Se to alleviate kidney damage triggered by heightened ER stress, oxidative stress, and apoptosis in rats fed with HFCS. Our findings revealed that Se effectively mitigated histological alterations, ER stress, oxidative stress, and apoptotic mechanisms in HFCS-induced kidney injury.

## Data Availability

It can be obtained from the corresponding author upon request.
